# LncRNA HAR1A Suppresses the Development of Non-Small Cell Lung Cancer by Inactivating the STAT3 Pathway

**DOI:** 10.3390/cancers14122845

**Published:** 2022-06-08

**Authors:** Jianqun Ma, Kui Cao, Xiaodong Ling, Ping Zhang, Jinhong Zhu

**Affiliations:** 1 Department of Thoracic Surgery, Harbin Medical University Cancer Hospital, 150 Haping Road, Harbin 150040, China; jianqunma@hrbmu.edu.cn (J.M.); lxdhmu@163.com (X.L.); 2 Department of Clinical Laboratory, Biobank, Harbin Medical University Cancer Hospital, 150 Haping Road, Harbin 150040, China; caokui19940505@gmail.com (K.C.); zhang15587333750@163.com (P.Z.); 3 Department of Clinical Oncology, Harbin Medical University Cancer Hospital, 150 Haping Road, Harbin 150040, China

**Keywords:** lncRNA *HAR1A*, STAT3, proliferation, prognostic signature, LUAD

## Abstract

**Simple Summary:**

We found that lncRNA Highly Accelerated Region 1A (*HAR1A*) was down regulated in NSCLC. Moreover, a 23-gene signature derived from *HAR1A*-related cancer cell survival genes could predict prognosis and chemotherapy response in LUAD. In vitro experiments indicated that *HAR1A* suppressed NSCLC growth by inhibiting the STAT3 signaling pathway, which was verified in the animal model. Overall, HAR1A acts as a tumor suppressor in NSCLC. The prognostic signature showed promise in predicting prognosis and chemotherapy sensitivity.

**Abstract:**

It is imperative to advance the understanding of lung cancer biology. The Cancer Genome Atlas (TCGA) dataset was used for bioinformatics analysis. CCK-8 assay, flow cytometry, and western blot were performed in vitro, followed by in vivo study. We found that lncRNA *Highly Accelerated Region 1A* (*HAR1A*) is significantly downregulated in lung adenocarcinoma (LUAD) and negatively associated with prognosis. We improved the prognostic accuracy of *HAR1A* in LUAD by combining genes regulating cell apoptosis and cell cycle to generate a 23-gene signature. Nomogram and decision curve analysis (DCA) confirmed that the gene signature performed robustly in predicting overall survival. Gene set variation analysis (GSVA) demonstrated several significantly upregulated malignancy-related events in the high-risk group, including DNA replication, DNA repair, glycolysis, hypoxia, MYC targets v2, and mTORC1. The risk signature distinguished LUAD patients suitable for chemotherapies or targeted therapies. Additionally, the knockdown of *HAR1A* accelerated NSCLC cell proliferation but inhibited apoptosis and vice versa. *HAR1A* regulated cellular activities through the STAT3 signaling pathway. The tumor-suppressing role of *HAR1A* was verified in the mouse model. Overall, the gene signature was robustly predictive of prognosis and sensitivity to anti-tumor drugs. *HAR1A* functions as a tumor suppressor in NSCLC by regulating the STAT3 signaling pathway.

## 1. Introduction

Non-small cell lung cancer constitutes 85% of all lung cancer, the top cause of cancer-related deaths [1]. Over the past decade, striking progress has been made in the treatment paradigm for NSCLC, including targeted therapy and immunotherapy-based treatments [2]. For instance, several tyrosine kinase inhibitors, targeting EGFR, ALK, RET receptor tyrosine kinase (RET), and proto-oncogene tyrosine-protein kinase ROS (ROS1), are the standard of care for lung adenocarcinoma (LUAD) patients carrying corresponding molecular mutations [1,2,3,4]. However, fewer than a quarter of NSCLC patients benefit from targeted therapy, and drug resistance often occurs in patients initially sensitive to the treatment [5]. Moreover, for the majority of NSCLCs lacking druggable driver molecular alterations, combination chemotherapy regimens remain the first option by far, with a median overall survival (OS) of less than two years for advanced or metastatic diseases [6]. Lung cancer is a disease with great molecular heterogeneity. It is imperative to understand its biology to develop more effective therapies for patients with different molecular characteristics.

Long non-coding RNAs (lncRNAs) are transcribed RNAs consisting of more than 200 nucleotides, lacking protein-coding potential [7]. LncRNAs were neglected before but are now known to regulate various fundamental biological processes, including development, differentiation, metabolism, and carcinogenesis. The expression of these non-coding molecules seems to be stringently regulated in physiological conditions but dysregulated in cancer. Great attention has been paid to the regulatory implications of lncRNA in NSCLC. Overexpressed LINC00173.v1 in lung squamous cell carcinoma upregulated VEGFA, promoting angiogenesis and tumor progression [8]. The upregulation of LINC01234 was associated with increased metastasis and shortened survival in NSCLC [9]. Gupta et al. demonstrated that lncRNA ANRIL and UFC1 were promising druggable targets for NSCLC [10]. The tumor-suppressing lncRNAs are also reported. Ma et al. reported that lncRNA GAS5 inhibited the progression of NSCLC by competitively preventing interaction between miR-221-3p and IRF2 [11]. Moreover, AFAP1-AS1 deficiency was shown to facilitate NSCLC growth in vitro and in vivo [12]. Collectively, the growing connections between non-coding RNAs and cancer have heralded that lncRNAs may function as biomarkers or therapeutic targets for cancer.

By exploiting public databases, we find that lncRNA *Highly Accelerated Region 1A* (*HAR1A*), mapped to chromosome 20q13.33, was significantly less abundant in cancerous tissues than normal tissues and negatively associated with overall survival in LUAD. There are very few publications regarding this molecule to date, but several studies have suggested *HAR1A* as a tumor suppressor in oral cancer [13], hepatocellular carcinoma (HCC) [14], and diffuse glioma [15]. In this study, we performed bioinformatic analysis, followed by in vitro and in vivo invalidations to explore the implications of this lncRNA in LUAD.

## 2. Materials and Methods

### 2.1. Bioinformatic Analysis

#### 2.1.1. Identification of Differentially Expressed LncRNA in LUAD

We first acquired the RNA-Seq data set and clinical information for The Cancer Genome Atlas (TCGA) lung adenocarcinoma cohort (https://nci.nih.gov/tcga/, accessed on 28 October 2020). We incorporated 59 normal and 517 tumor samples in the study after removing patients lacking clinical information. We also included a GSE40419 dataset consisting of RNA-Seq data for 87 lung adenocarcinomas and 77 adjacent normal tissues. The data were processed as described before [16,17,18]. We identified 65 downregulated and 95 upregulated intergenic lncRNAs (lincRNAs) in LUAD relative to normal tissues (log2FC > 0.4 or <−0.4, adjusted *p*-value < 0.05). Among these lincRNAs, lncRNA *HAR1A* was associated with survival and selected for further study. Expression levels of this lncRNA were compared between tumor and normal tissues across 33 types of cancers using the TCGA database. 

#### 2.1.2. Established a Prognostic Signature from HAR1A-Related Apoptosis and Cell Cycle Genes

In brief, 13,465 lncRNA *HAR1A*-related genes were acquired from the TCGA-LUAD cohort using Pearson’s correlation analysis, constituting gene list 1. Next, we retrieved 161 apoptosis- and 125 cell cycle-related genes from GSEA datasets forming gene list 2. Then, 274 genes common to both lists were named *HAR1A*-related apoptosis and cell cycle genes. These genes were screened using univariable Cox regression analysis for OS, and genes significantly associated with survival were retained for the study.

The least absolute shrinkage and selection operator (LASSO)–penalized multivariable Cox regression was used to build a prognostic model for OS. The remaining survival genes were entered into a LASSO regression model, and genes with non-zero coefficients were used to establish a multi-gene survival model. The resulting LASSO coefficients and corresponding patient expression levels of each included gene were incorporated into a risk score algorithm to quantitatively evaluate patient risk as published previously [16,19,20]. To assess the predictive ability of the prognostic multiple gene models, time-dependent receiver operating characteristic (ROC) curves were plotted for the risk scores with the utilization of the R package survivalROC. The area under the ROC curve (AUC) was calculated and used as a metric of discriminatory ability. The maximum Youden’s J statistic (J = sensitivity + specificity − 1) on the time-dependent ROC curve was used as a cutoff to separate patients into high- and low-risk groups. A Kaplan–Meier curve was plotted for the risk score-defined groups with the R package rms, followed by the log-rank test. Uni- and multivariable Cox regression analyses were employed to evaluate the possible predictors of OS, including clinical features and the risk scores.

#### 2.1.3. Construction of a Nomogram

Furthermore, we constructed a Cox-based nomogram for the individual prediction of the OS. We also plotted the observed against the expected OS to build a calibration curve. Moreover, to assess the potential impact of the nomograms on clinical management, decision curve analysis (DCA) was performed, which helped determine whether the models were clinically beneficial by generating the net benefit. 

#### 2.1.4. Prediction of Drug Sensitivity Using the Risk Model

We also examined whether the risk model can facilitate the prediction of drug sensitivity in LUAD patients. We calculated the log (half inhibitory concentration (IC50)) of several common administrating chemotherapeutic drugs in the LUAD cohort by utilizing an R package pRRophetic [21,22]. It is an R package that is able to predict clinical response to 138 drugs by integrative analysis of tumor gene expression profiles in patients. In this study, anti-tumor drugs such as paclitaxel, gemcitabine, cisplatin, and docetaxel were investigated in the TCGA-LUAD cohort. Wilcoxon signed-rank test was performed to check the difference in the log (IC50) between the high and low-risk groups.

#### 2.1.5. Gene Set Variation Analysis for Underlying Molecular Features

We carried out gene set variation analysis (GSVA) to reveal the molecular mechanisms under the different risk groups using the GSVA package for R. Gene signatures were acquired from HALLMARK and KEGG databases. 

### 2.2. Cell Culture

Normal human lung bronchial epithelial (BEAS-2B) and three human NSCLC cell lines, including NCI-H1975, NCI-H292, and A549, were obtained from ATCC, while PC-9 and PLA-801D were from BCRJ and CCTCC, respectively. Cells were regularly passaged in RPMI-1640 or DMEM medium (Procell, Wuhan, China) with the addition of penicillin G (100 U/mL, NCM Biotech, Suzhong, China), streptomycin (100 μg/mL, Corning Incorporated, Corning, NY, USA), and 10% fetal bovine serum (Hyclone, Life Sciences, Shanghai, China). The cultures of NSCLC cells were kept in an incubator (Thermo, Waltham, MA, USA) under standard temperature and humidity as well as 5% CO_2_ and 95% air. 

“Loss of function” of *HAR1A* was implemented using a lentivirus system, pLVX-shRNA-puro, delivering shRNAs targeting lncRNA *HAR1A*, as well scrambled sequence used as control. Sequences were listed as follows: Ctrl 5′-TTCTCCGAACGTGTCACGT-3′, *HAR1A* 5′GCATGTGTAACATCAACAT-3′. Meanwhile, the *HAR1A* overexpression lentivirus system was also constructed. We cloned the coding DNA sequence (CDS) region of *HAR1A* (NR_003244.2), synthesized by Genewiz Company (Shanghai, China), into the EcoR I/BamHI restriction sites of a pLVX-IRES-ZsGreen1 vector. In order to generate lentivirus to manipulate gene expression, pLVX -sh *HAR1A* -Puro or pLVX-IRES-ZsGreen1-*HAR1A* were co-transfected with psPAX2 and pMD2.G (Addgen, Watertown, MA, USA) viral packaging plasmids into 293 cells with the use of Lipofectamine 2000 (Thermo Fisher Scientific, Waltham, MA, USA). After 48 h of infection, the lentivirus particles were collected and stored. While reaching 80% confluency, cells were infected with different lentiviruses with a multiplicity of infection (MOI) of 50.

### 2.3. RNA Extraction and Quantitative Reverse Transcription—PCR (qRT-PCR)

While cells reached 75% confluency in 75 cm^2^ flasks, we removed the culture medium and extracted total RNA using TRIzol (Thermo Fisher Scientific, Waltham, MA, USA). The concentration of RNA samples was determined with a Nanodrop1000 spectrophotometer (NanoDrop, Madison, WI, USA). Then, 1 μg of RNA was reverse transcribed into complementary DNA (cDNA) using SureScript™ First-Strand cDNA Synthesis Kit (Genecopoeia, Guangzhou, China). The reverse transcription primer mix contains a specially optimized mix of oligo-dT and random primers that enable cDNA synthesis from all regions of RNA transcripts.

The qPCR assays were performed on a *qTOWER* 3 Real-Time PCR Thermal Cyclers (Analytik Jena AG, Germany) with 20 μL of reaction volumes comprising 10 μL of 2x BeyoFast™ SYBR Green qPCR Mix (Beyotime, Shanghai, China), 2 μL of primers.

(Forward Primer: 5′ ACTCTGGTGTGTCCCGTTTGAA 3′ and Reverse primer: 5′ TCTGTGTGTTGCCACCTCCG 3′), 2 μL of cDNA template, and 6 μL of ddH_2_O. The thermal cycle protocol used was as follows: 50 °C for 2 min, 10 min initial denaturation at 95 °C, and 40 cycles of 15 s denaturation at 95 °C, 30 s annealing at 60–68 °C. GAPDH was used as a housekeeping gene for all the qPCR experiments (Forward Primer: 5′ ACAGCCTCAAGATCATCAGC 3′ and Reverse primer: 5′ GGTCATGAGTCCTTCCACGAT 3′). Relative gene expression was calculated using the comparative CT method known as 2^−ΔΔCt^. Expression analyses in cells were normalized to GAPDH. 

### 2.4. Cell Proliferation Assay

Cell counting kit-8 (CCK-8; Dojindo Molecular Technologies, Rockville, MD, USA) was adopted to examine cell proliferation. NSCLC cells were seeded in 96-well culture plates at a density of 5 × 10^3^ cells/well in triplicate, and then incubated in a 5% CO_2_ humidified incubator at 37 °C overnight. At each indicated time point, we removed the medium and added 100 μL 10% CCK-8 reagent to each well, followed by incubation for an additional 2 h at 37 °C. Finally, cell proliferation was measured as absorbance at the wavelength of 450 nm using an ELISA plate reader.

### 2.5. Cell Apoptosis Analysis

Apoptotic cells were labeled with the Annexin V-FITC/PI Apoptosis Detection Kit I (BD Biosciences, San Jose, CA, USA) and quantified on flow cytometry. Briefly, cells were harvested using trypsin/EDTA and washed with PBS. After centrifuge, cell pellets were re-suspended in the staining buffer with the addition of FITC-labeled Annexin V and PI and kept in the dark for 10–15 min at room temperature. Finally, the apoptotic cells were detected by a FACScan Flow Cytometer (Becton Dickinson, Franklin lakes, NJ, USA). We also studied whether lncRNA *HAR1A* regulates the proliferation of NSCLC cells through the STAT3 signaling pathway. Cells infected with lenti-sh *HAR1A* were treated with Stattic, an inhibitor of STAT3 (MedChemExpress LLC, Princeton, NJ, USA), for 24 h.

### 2.6. Gene Set Enrichment Analysis to Dissect Downstream Pathways of HAR1A

The RNAseq data of 119 NSCLC cancer cell lines were retrieved from the Cancer Cell Line Encyclopedia (CCLE) database (https://sites.broadinstitute.org/ccle, accessed on 14 November 2020). The median expression level of the lncRNA *HAR1A* gene was used to divide all NSCLC cell lines into lncRNA *HAR1A*^high^ and lncRNA *HAR1A*^low^ groups. GSEA software 4.0.0 was utilized to analyze the lncRNA *HAR1A*-related signaling pathways.

### 2.7. Western Blot

Cells were harvested and lysed using a cocktail of RIPA lysis buffer. The soluble protein concentration of cell lysate was read using Bradford reagent (Bio-Rad Laboratories, Hercules, CA, USA). Western blot analysis was performed following standard protocol. Generally, 10–20 μg of protein were loaded and separated in SDS-PAGE. The primary antibodies used for this study were as follows: antibodies against Bax (Abcam, Fremont, CA, USA), Bcl-2 (Abcam, Fremont, CA, USA), MCM2 (Proteintech, Wuhan, China), PCNA (Proteintech, Wuhan, China), GAPDH (Cell signaling Technology, Danvers, MA, USA), p-STAT3 (Cell Signaling Technology, Danvers, MA, USA), and STAT3 (Cell Signaling Technology, Danvers, MA, USA). Secondary HRP-conjugated antibodies against mouse or rabbit (Sigma-Aldrich, St. Louis, MO, USA) was used as proper. Protein bands were detected by Enhanced Chemiluminescence (Thermo Fisher Scientific, Waltham, MA, USA). The whole western blots are shown in File S1.

### 2.8. In Vivo Tumorigenicity Assay

The animal studies were reviewed and approved by the Harbin Medical University Cancer Hospital’s Institutional Review Board. Female BALB/c nude mice at 5–6 weeks of age were used in the experiments (SLAC laboratory animal lnc., Shanghai, China). Mice were housed under specific pathogen-free conditions and randomly divided into control and experimental groups. A549 cells transfected with LV-*HAR1A* (5 × 10^6^) suspended in growth factor-reduced Matrigel (Corning Incorporated, Corning, NY, USA.) were directly into the left upper flank regions of nude mice, while mice receiving the same number of A549-vector cells were considered as controls. Tumors were observed around a week after injection. Since the eighth day, tumor width and length were measured every five days with digital calipers (ProSciTech Pty Ltd., Kirwan, QLD, Australia), and tumor volume was calculated using the formula below: tumor volume = (length × width^2^) × 0.5. Mice were sacrificed at 28 days post-injection, and subcutaneous tumor tissues were harvested and weighted. Part of tumor tissues was used to extract total RNA to measure expression levels of *HAR1A*. The rest of the tissues were formalin-fixed, paraffin-embedded, and serially sectioned to examine the expression of minichromosome maintenance complex component 2 (MCM2) and proliferating cell nuclear antigen (PCNA) by immunohistochemistry (IHC) staining. Primary antibodies for IHC staining were purchased from Proteintech (Wuhan, China).

### 2.9. Statistical Analysis

Statistical analysis was carried out using the SPSS version 22 (SPSS, Inc., Chicago, IL, USA). The experimental results in vitro and in vivo were presented as the mean ± standard deviation (SD). The Student’s *t*-test was used to analyze differences between groups. For comparisons between multiple groups, a one-way analysis of variance (ANOVA) was performed, followed by Tukey’s multiple comparisons tests in order to achieve means separation. Differences were considered statistically significant at *p* < 0.05.

## 3. Results

### 3.1. Differentially Expressed LncRNAs in Lung Adenocarcinoma

**Figure 1:**
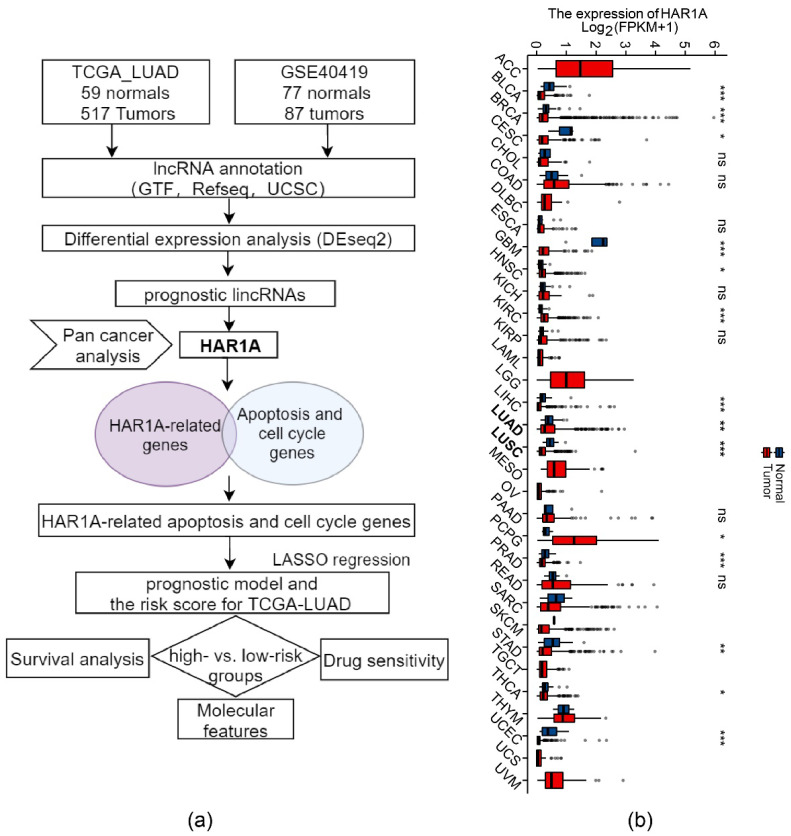
Decreased lncRNA HAR1A in tumors. (**a**) The workflow for selecting differentially expressed lncRNAs between LUAD and normal tissues. (**b**) lncRNA *HAR1A* was downregulated in various types of cancer. * *p* < 0.05, ** *p* < 0.01, *** *p* < 0.001, ns, not significant.

The workflow of bioinformatic analysis is shown in Figure 1a. We first identified 65 downregulated and 95 upregulated intergenic lncRNAs (lincRNAs) in LUAD relative to normal tissues (log2FC > 0.4 or <−0.4, adjusted *p*-value < 0.05) (Supplemental Figure S1b,c) by analyzing transcriptome sequencing data from The Cancer Genome Atlas (TCGA) project (Supplemental Figure S1a,b). LncRNAs were prioritized based on the degree of deregulation and potential clinical relevance. Among these lincRNAs, lncRNA *HAR1A* was also significantly decreased in the GSE40419 LUAD cohort (Supplemental Figure S1c,d). Kaplan–Meier survival analysis indicated that low levels of lncRNA *DHAR1A* were significantly associated with poor prognosis in the TCGA-LUAD cohort, which was also verified using KM plotter (http://kmplot.com/analysis/, accessed on 5 January 2020) (Supplemental Figure S1e,f). Moreover, the *HAR1A* levels were decreased in multiple cancer types, suggesting its universal importance in tumorigenesis Figure 1b).

### 3.2. Integrating Apoptosis and Cell Cycle-Related Genes to Improve the Prognostic Robustness of LncRNA HAR1A

**Figure 2:**
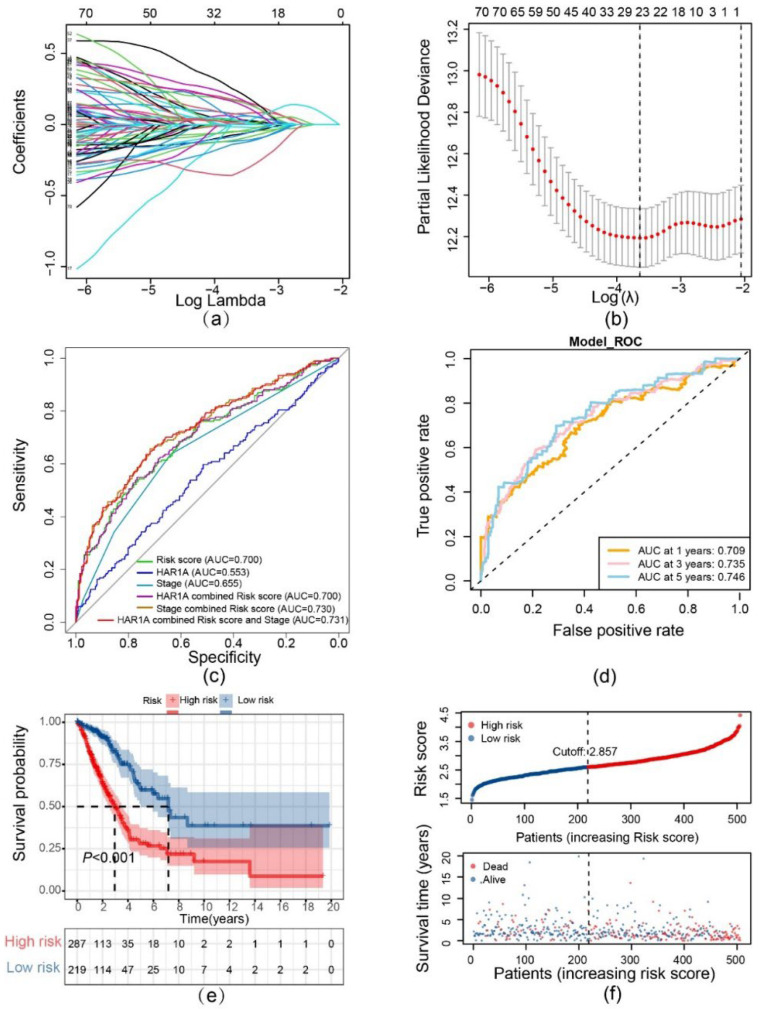
Construction and evaluation of the prognostic multiple-gene signature. (**a**) LASSO Cox regression coefficients of lncRNA *HAR1A*-related apoptosis/cell cycle genes. (**b**) Identification of the favorable gene signature using the LASSO model. (**c**) ROC curve analyses of the prognostic model, TNM stage, *HAR1A*, and indicated combinations. (**d**) Time-dependent receiver operator characteristic (ROC) curves trained on the risk scores were used to access AUC values for the TCGA-LUAD cohort, evaluating the overall accuracy of the model. (**e**) Kaplan–Meier survival curves of overall survival (OS) time between high- and low-risk patients. (**f**) The distribution of the risk score and survival status indicates that the survival time of patients descends while the risk scores are increasing.

Given the universal importance of cell proliferation and apoptosis in tumorigenesis, we seek to enhance the prognostic accuracy of this lncRNA by integrating crucial genes controlling apoptosis and the cell cycle. In the end, LASSO regression yielded the best predictive signature composed of 23 genes with non-zero regression coefficients (Figure 2a,b; Table 1). A risk score was calculated for each patient based on the gene signature [16,17,18]. As shown in the receiver operating characteristic (ROC) curves, the risk score achieved a decent prediction accuracy with an area under the curve (AUC) of 0.700 in comparison to AUCs of 0.553 and 0.655 for the *HAR1A* and TNM stages, respectively. Prediction accuracy (AUC = 0.731) culminated while *HAR1A* and stage were incorporated into this risk model (Figure 2c). The time-dependent ROC analysis revealed an AUC for the risk score as high as 0.746 at five years (Figure 2d). Furthermore, the high-risk group defined by the risk score showed an inferior prognosis when compared to the low-risk group (*p* < 0.001) (Figure 2e), and fewer patients survived in the high-risk group than in the low-risk group (Figure 2f). 

**Figure 3:**
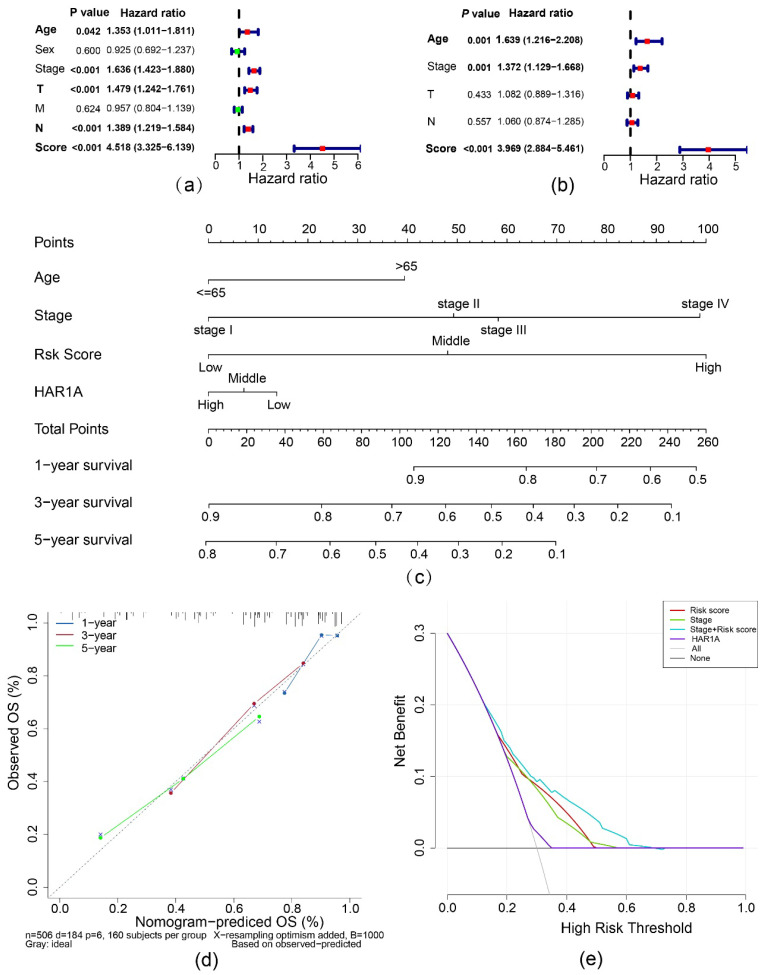
Evaluation of the prognostic value of risk score. (**a**) Univariate analysis to dissect the factors significantly associated with OS. (**b**) Multivariate analysis to determine the independent prognostic factors. (**c**) Nomogram for the prediction of 3- and 5-year survival. (**d**) Calibration curves illustrate the concordance between predicted and actual survival possibilities within 3 and 5 years. (**e**) Decision curve analysis unveils that the combination of risk score and TNM stage is superior to TNM stage alone across threshold probabilities of survival between 18% and 80%. The grey and blue lines depict a ‘treat none’ strategy and ‘treat all’ strategy, respectively.

Multivariable Cox regression analyses, adjusting for age, stage, T, and N, demonstrated that risk score remained the most critical predictor of OS (adjusted hazard ratio (HR) = 3.969, 95% confidence interval (CI) = 2.884–5.461, *p* < 0.001) (Figure 3a,b). The clinical impacts of different variables in predicting 1-, 3- and 5-year OS rates were visualized in the nomogram with a decent concordance index of 0.724 in the TCGA-LUAD cohort (Figure 3c,d). The risk score improved decision-making compared with stage, and net benefits were further enhanced by combining risk score and stage (Figure 3e). 

### 3.3. Prediction of Patient Response to Chemotherapies

**Figure 4:**
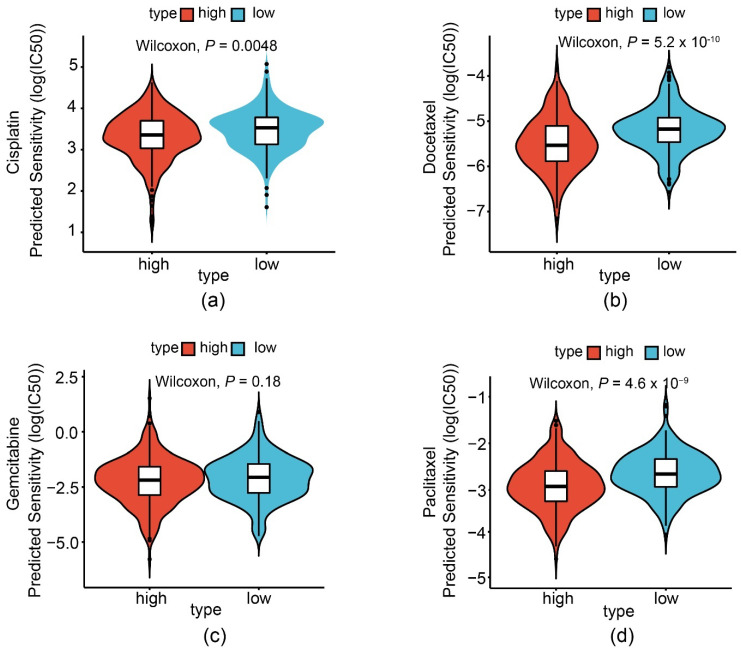
The risk score predicts the sensitivity of the TCGA-LUAD patients to chemotherapy. Predicted response to drugs, including cisplatin (**a**), docetaxel (**b**), gemcitabine (**c**), and paclitaxel (**d**).

Next, we tried to test the predictive ability of the risk score on drug sensitivity in LUAD. Using the pRRophetic methodology, we found that the high- and low-risk groups significantly differed in sensitivity to cisplatin, paclitaxel, and docetaxel, as indicated by the log (IC50) (Figure 4a–d). 

### 3.4. Underlying Molecular Features between High- and Low-Risk Groups

**Figure 5:**
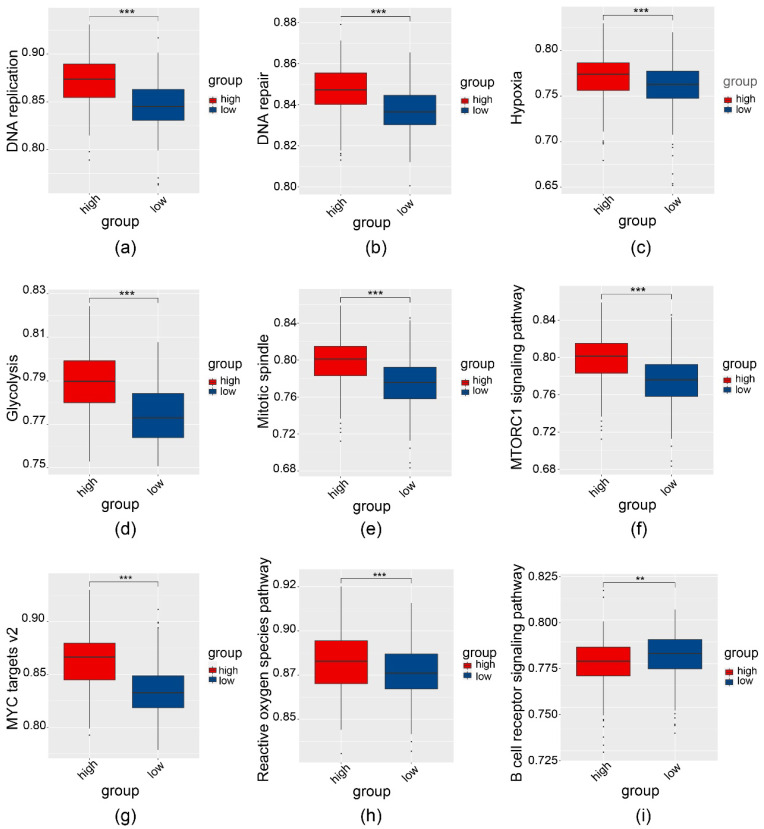
Gene set variation analysis to compare key molecular features between high- and low-risk groups. Significant differences in cellular and molecular features between the two groups are as follows: (**a**) DNA replication, (**b**) DNA repair, (**c**) hypoxia, (**d**) glycolysis, (**e**) mitotic spindle, (**f**) mTORC1 signaling pathway, (**g**) MYC targets v2, (**h**) reactive oxygen species pathway, and (**i**) the B cell receptor pathway. ** *p* < 0.01, *** *p* < 0.001.

Regarding molecular and cellular characteristics, GSVA unveiled that several malignancy-related events were significantly altered in the high-risk group compared with the high-risk group (Figure 5a–i).

### 3.5. Expression of LncRNA HAR1A in NSCLC Cell Lines

**Figure 6:**
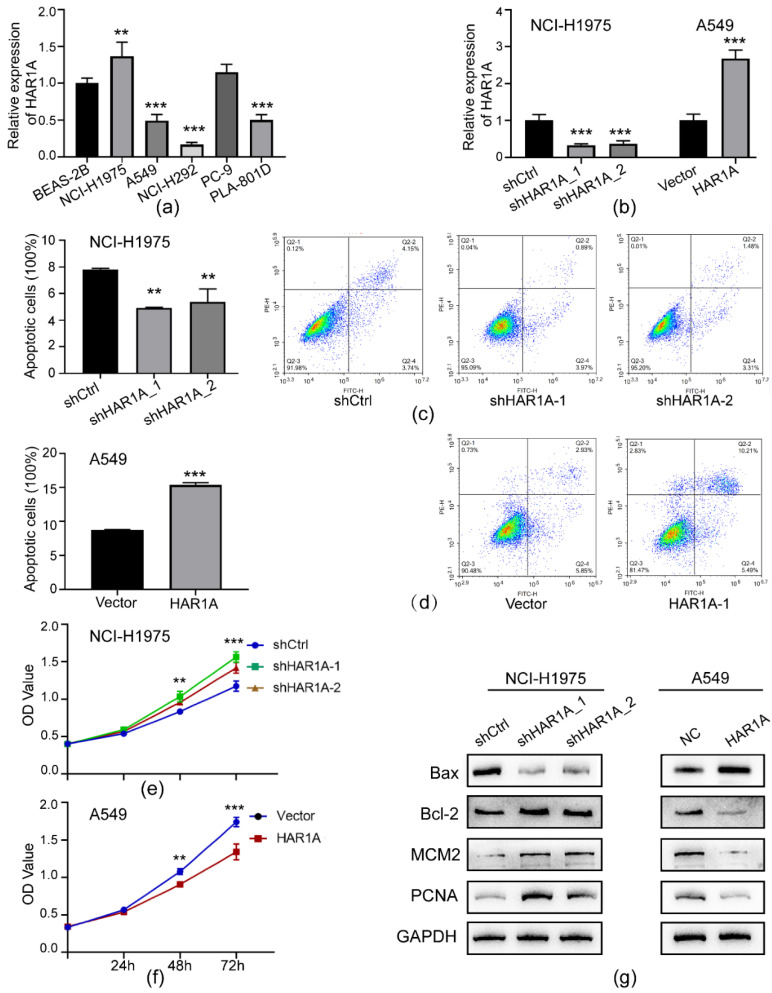
LncRNA *HAR1A* promoted apoptosis and inhibited the proliferation of NSCLC cells. (**a**) The expression levels of lncRNA *HAR1A* in five NSCLC cell lines as well as normal lung epithelial cells by qRT-PCR. (**b**) qRT-PCR results confirmed loss or gain function of lncRNA HAR1A in NCI-1975 and A549 cells. Impacts of the knockdown (**c**) of overexpression (**d**) of lncRNA *HAR1A* on apoptosis of NSCLC cells. After Annexin V and PI double staining, the percentage of apoptotic cells was estimated using flow cytometry. (**e**) The growth curve of A549 cells infected with lentivirus-mediated shRNA oligos targeting lncRNA *HAR1A* or scramble sequences (Ctrl). (**f**) The growth curve of A549 cells infected with empty vector (Ctrl) or lentivirus-mediated lncRNA *HAR1A*. Cell proliferation was determined by CCK-8 assay at 24, 48, and 72 h. (**g**) The expression of apoptotic (BAX and BCL-2) and proliferative (MCM2 and PCNA) biomarkers were examined in NSCLC cells by western blot. The experiment was performed in triplicate, and data were calculated from three independent experiments using statistical analysis. ** *p* < 0.01, *** *p* < 0.001.

Given the potential clinical relevance of lncRNA *HAR1A* revealed by the bioinformatic analysis above, we further performed functional studies on this molecule. We first compared the expression of *HAR1A* in NSCLC and normal cell lines by qRT-PCR. As illustrated in Figure 6a, the expression levels of lncRNA *HAR1A* were significantly upregulated in NCI-1975 but downregulated in A549, NCI-H292, and PLA-801D NSCLC cells when compared with BEAS-2B cells. To evaluate the effects of lncRNA *HAR1A* on NSCLC cell proliferation, lncRNA *HAR1A* was knocked down in NCI-H1975 cells using specific lentiviral shRNA and overexpressed in A549 cells using a lentiviral vector. Infection of LV-sh*HAR1A* significantly reduced the expression level of lncRNA *HAR1A in* NCI-H1975 cells, whereas *HAR1A* expression was significantly elevated in LV-*HAR1A* infected A546 cells (Figure 6b). 

### 3.6. LncRNA HAR1A Promoted Apoptosis and Inhibited the Proliferation of NSCLC Cells

Resisting cell death is one of the cancer hallmarks. Therefore, we investigated the potential influences of lncRNA *HAR1A* on the apoptosis of NSCLC cells. After sh*HAR1A_1* and sh*HAR1A_2* lentivirus infection, only 4.91% and 5.36% of NCI-H1975 cells underwent apoptosis in comparison to 7.81% apoptotic cells detected in shCtrl lentivirus infected cells (Figure 6c). Moreover, our results confirmed that overexpression of lncRNA *HAR1A* significantly promoted cell apoptosis (Figure 6d). We then evaluated the effect of lncRNA *HAR1A* on NSCLC cell proliferation. CCK-8 assays demonstrated that ablation of lncRNA *HAR1A* expression significantly accelerated the proliferation of NCI-1975 cells compared with controls (Figure 6e). In contrast, cell proliferation was significantly suppressed in A549 cells with lncRNA *HAR1A* overexpression (Figure 6f). Correspondingly, WB analysis revealed that silencing of lncRNA *HAR1A* increased Bcl-2, which inhibited cell apoptosis, and decreased pro-apoptotic Bax while increasing proliferative biomarkers, MCM2 and PCNA (Figure 6g, Supplemental Figure S2) and vice versa. These results suggest that lncRNA HAR1A may be a tumor-suppressing gene in NSCLC, promoting apoptosis and inhibiting the proliferation of NSCLC cells.

### 3.7. LncRNA HAR1A Regulated STAT3 Signaling Pathway in NSCLC Cells

**Figure 7:**
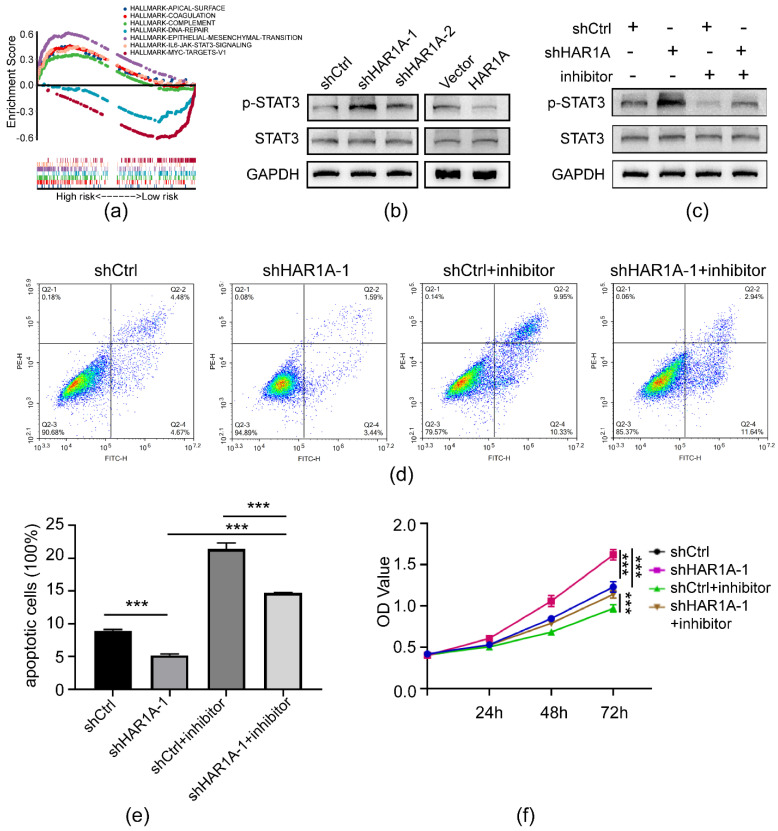
LncRNA *HAR1A* regulated NSCLC cell proliferation and apoptosis via the STAT3 signaling pathway. (**a**) GSEA revealed that the IL-6-JAK-STAT3 pathway was associated with lncRNA *HAR1A* in NSCLC cell lines. (**b**) Western blot was used to detect expression and phosphorylation levels of STAT3 under the influence of the knockdown or overexpression of lncRNA *HAR1A*. (**c**) shCtrl and sh*HAR1A* cells and controls were treated with or without STAT3 inhibitor, Stattic. Western blot was performed to examine the levels of the indicated proteins. (**d**,**e**) Cell apoptosis was determined by flow cytometry. (**f**) Cell proliferation was measured by CCK8. The experiments were performed in triplicate, and data were calculated from three independent experiments using statistical analysis. *** *p* < 0.001.

To further elucidate the underpinning mechanism by which lncRNA *HAR1A* promotes NSCLC tumorigenesis, we conducted GSEA to screen the potential downstream signaling pathway in lung adenocarcinoma with TCGA datasets. Briefly, we analyzed the coexpression genes of *HAR1A* with Spearman correlation to obtain the *HAR1A*-related gene network. The clusterProfiler package for R was used to perform GSEA analysis on these genes. As a result, *HAR1A*-related genes were enriched in the G2/M cell cycle checkpoints, interferon-gamma/alpha response, Wnt/catenin pathway, TP53 pathway, oxidative phosphorylation, cellular inflammatory response, and other processes (Figure 7a). These pathways all play a critical role in the initiation and development of lung adenocarcinoma.

Next, we investigated the effects of lncRNA HAR1A on the STAT3 signaling pathway in NSCLC cells. In agreement with the results of bioinformatics analysis, western blot analysis showed that phosphorylation levels of STAT3 were decreased by overexpression of lncRNA *HAR1A* but enhanced by the silencing of the same lncRNA molecule (Figure 7b, Supplemental Figure S3). These data indicate that lncRNA *HAR1A* may inactivate the STAT3 signaling pathway in NSCLC. 

### 3.8. LncRNA HAR1A Mediated the Proliferation and Apoptosis of NSCLC Cells via the STAT3 Signaling Pathway

To verify whether the STAT3 signaling pathway is indispensable for lncRNA *HAR1A*-mediated NSCLC cell proliferation in vitro, we treated NSCLC cells infected with lenti-sh*HAR1A* with a STAT3 inhibitor. As indicated in Figure 7c and Supplemental Figure S4, the STAT3 inhibitor treatment alone decreased the level of p-STAT3 in NCI-1975 cells and rescued the effects of sh*HAR1A*. Interestingly, we found that inhibition of the STAT3 signaling pathway reversed the effects of lncRNA *HAR1A* knockdown on proliferation and apoptosis of NSCLC cells. As shown in Figure 7d,e, silencing of lncRNA *HAR1A* inhibited cell apoptosis, which was restored during incubation with STAT3 inhibitor. Moreover, *HAR1A* knockdown-induced cell proliferation was abolished by inhibiting STAT3 (Figure 7f). Overall, rescue experiments confirm that the STAT3 signaling pathway is responsible for the tumor-suppressing effects of lncRNA *HAR1A* in NSCLC.

### 3.9. LncRNA HAR1A Overexpression Inhibited the Proliferation of NSCLC In Vivo

**Figure 8:**
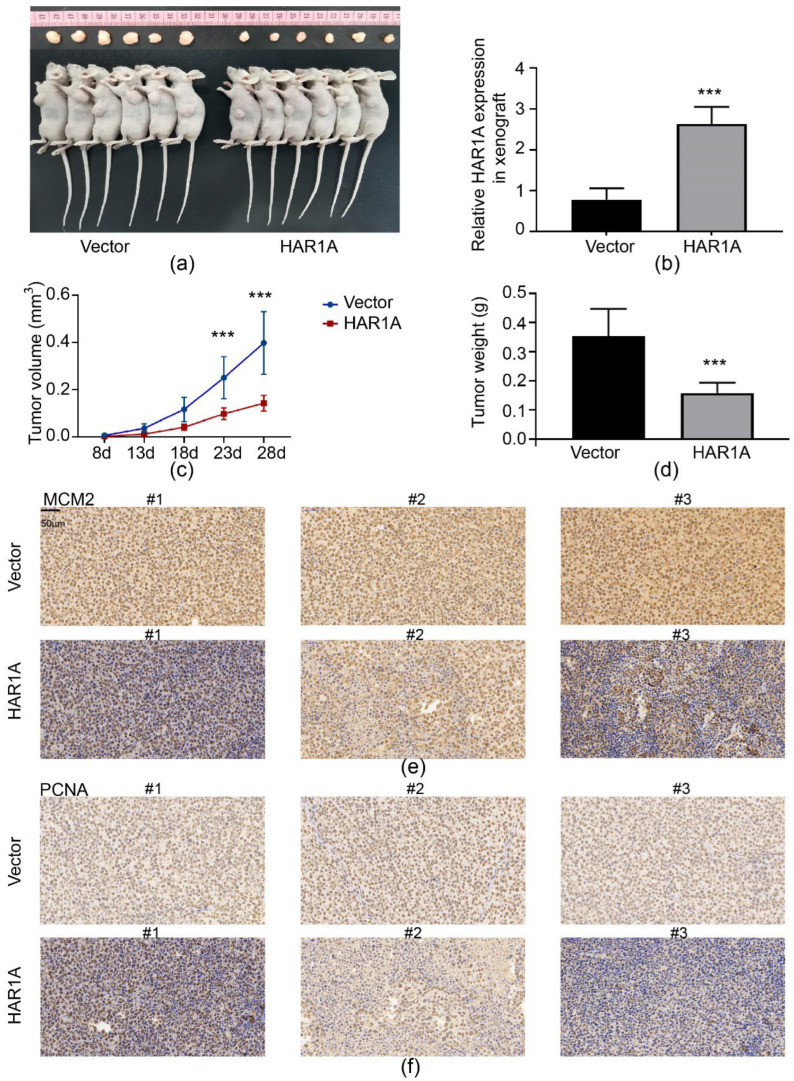
Tumor-suppressing effects of lncRNA *HAR1A* on NSCLC in vivo. (**a**) Images of tumors in nude mice generated from A549 cells transfected with LV-NC and LV-*HAR1A*. (**b**) qRT-PCR analysis of lncRNA HAR1A levels in two groups of nude mice. Comparison of tumor volumes (**c**) and tumor weights (**d**) between LV-NC and LV-*HAR1A* groups (n = 6). (**e**) Immunohistochemical staining of MCM2 on tumors for LV-NC and LV-*HAR1A* groups (n = 3). (**f**) Immunohistochemical staining of PCNA on tumors for LV-NC and LV-*HAR1A* groups, *** *p* < 0.001.

Given the in vitro results, we further verified the tumor-suppressing role of *HAR1A* in NSCLC by using a xenograft tumor model. We first generated xenograft tumors by subcutaneously injecting A549 cells infected with lenti-NC or lenti-*HAR1A* into nude mice. As shown in Figure 8a, xenogeneic tumors were observed at the injection site in all nude mice. However, overexpression of *HAR1A* seemed to lead to a decrease in tumor size. The qRT-PCR analysis confirmed significantly upregulated *HAR1A* expression levels in lenti-*HAR1A* cell-derived tumors when compared to those in control tumors (Figure 8b). A statistical study indicated that tumors with overexpressed *HAR1A* grew slower than tumors from control cells (Figure 8c). *HAR1A* enforced expression also significantly downregulated the weight of xenograft tumors in mice (Figure 8d). IHC staining unveiled that overexpression of *HAR1A* reduced tumor cell proliferation, as shown by decreased MCM2 and PCNA positive tumor cells in lenti-*HAR1A* cell-derived tumors (Figure 8e,f). Overall, these results suggest that the knockdown of *HAR1A* inhibited NSCLC cell growth in vivo.

## 4. Discussion

NSCLC is a leading cancer and also one of the deadliest malignant tumors in the world. Irrespective of the tremendous progress made in multimodality therapy, including surgery, chemotherapy, radiotherapy, targeted therapy, and immunotherapy, the five-year overall survival of NSCLC remains disappointing [1,2,3]. NSCLC is a complicated multifaced disease. What we know about NSCLC to date is just the tip of the iceberg. Intensive and continuous efforts are warranted to clarify the potential mechanisms involved in tumorigenesis of NSCLC. Improved understanding of causal molecular aberrations may lead to novel biomarkers for early diagnosis, targeted treatment, and prognosis evaluation in NSCLC. Aberrant expression of lncRNAs is involved in tumorigenesis through various mechanisms, including competing for endogenous RNA, epigenetic modification, transcription regulation, and posttranslational regulation [7,23]. Abundant studies have shown that lncRNAs are crucial for lung cancer metastasis and prognosis [8,9,12,24,25]. For instance, aberrant upregulation of LINC01234 could elevate VAV3 and reduce BTG2 expression, thereby promoting NSCLC metastasis [9]. LINC00173.v1 aggravated angiogenesis and progression of lung squamous cell carcinoma by inhibiting miR-511-5p induced VEGFA degradation [8]. 

LncRNA GAS5 was also reported to modulate the progression of NSCLC by serving as competing endogenous RNA [11]. Recently, tumor-promoting roles of lncRNA ANRIL and lncRNA UFC1 were reported in NSCLC [10]. We identified a significantly differently expressed lncRNA *HAR1A* in LUAD using bioinformatics analysis, and the deficiency of *HAR1A* was significantly associated with poor clinical outcomes in the TCGA-LUAD cohort. Thus far, there are very few publications available regarding this molecule. *HAR1A* acted as a tumor suppressor for oral cancer by regulating the ALPK1/BRD7/myosin IIA axis in oral cancer [13]. Its tumor-suppressing potentials were also demonstrated in hepatocellular carcinoma (HCC) [14], and diffuse glioma [15].

In disproportion to the dramatic progress in cancer treatment, predicting cancer survival still largely relies on the American Joint Commission on Cancer Tumor–Node–Metastasis (TNM) staging system. However, failure to take molecular features of cancer into account unavoidably limits the predictive value of the traditional method. The inaccurate classification of patient risk for poor survival may mislead physicians into selecting inappropriate treatment regimens for cancer patients. Therefore, it is imperative to develop reliable and robust biomarkers for cancer prognosis to improve cancer management. Some molecular biomarkers predictive of progressive disease have been applied to select patients for suitable therapeutic interventions. For instance, mutation and expression of epidermal growth factor receptor (EGFR) were used to screen for the subpopulation of lung cancer patients for EGFR inhibitors, while PD-1/PD-L1 for checkpoint inhibitors. These current biomarkers are helpful but do not fully characterize the complex underlying mechanisms of cancer progression in lung cancer. To overcome this obstacle, dramatic advances in high-throughput mRNA profiling techniques and the blossoming of machine learning applications to RNA-seq data analysis have enabled the development of prognostic gene expression signatures. Over the past year, various types of gene signatures have been developed to improve the prediction of prognosis or therapeutic benefit [17,18,20,26,27,28,29,30]. For example, Shukla et al. reported an RNA-Seq Based Prognostic Signature in Lung Adenocarcinoma [30], and Li et al. developed a personalized immune prognostic signature for early-stage nonsquamous NSCLC [27]. 

However, it should be noted that gene function and its linkage to cancer development may provide useful fundamental information facilitating biomarker candidate screening [31]. In particular, practical information about gene regulation on cancer cell survival can disclose molecular implications for candidate biomarkers in cancer progression. Such valuable information cannot be derived from gene profiling analyses. Similar to other single biomarkers, LncRNA *HAR1A* only showed limited prognostic power and clinical efficacy (AUC = 0.553). One reason for the unsatisfying result is possibly the ignorance of the functional significance of biomarker genes to cancer development [31]. We built a predictive gene signature by incorporating gene expression profiles from TCGA-LUAD and critical proliferation and cell cycle gene datasets from GSEA. The resulting 23-gene signature could significantly stratify the TCGA-LUAD cohort into low- and high-risk groups, and statistics revealed that these two groups significantly differed in OS. ROC analysis demonstrated an AUC of 0.700 for the de novo prognostic gene signature. Moreover, the discrimination ability of the combination of the risk score, stage, and *HAR1A* compared favorably to a model with only each parameter alone (ROC = 0.731). Multivariate Cox regression analysis denoted the risk score to be an independent predictor of prognosis in LUAD. Moreover, this prognostic signature’s predictive accuracy and clinical benefit were further verified using nomogram and DCA. Others also succeeded in creating prognostic gene signatures by considering biological functions [28,31,32,33,34]. A predefined cell-cycle progression (CCP) gene signature, called CCP score, was shown to independently predict a 5-year risk of lung cancer-related death for early-stage lung adenocarcinoma [28,32]. Shen et al. separately identified the most ubiquitously expressed genes in LUAD from TCGA and GEO databases and essential survival genes from The Cancer Dependency Map (DepMap). Then, the two gene lists were intersected to identify cancer cell survival genes in lung cancer. By applying a backward stepwise variable regression model, they generated a 22-gene LUAD-progression gene signature, which demonstrated a robust performance in prognostic prediction [31]. Using the same method, Shen and colleagues also developed reliable gene PGSs for LUSC and glioblastoma, respectively [31]. Moreover, our team previously reported that UBE2T promoted autophagy in NSCLC, and a signature of 18 UBE2T-related autophagy genes greatly enhanced the predictive accuracy of prognosis compared to the UBE2T gene alone [19]. Taken together, these results suggest that genes critical for cancer cell survival are refined candidate biomarkers for prognostic prediction.

We also conducted functional analyses of lncRNA *HAR1A*. The results indicated that ectopic expression of *HAR1A* inhibited NSCLC cell proliferation in vitro and growth of NSCLC tumor xenograft and vice versa. Recently, Lee et al. reported that lncRNA *HAR1A* suppressed oral cancer progression [13]. Collectively, these indicate that *HAR1A* plays a tumor-suppressive role in tumorigenesis. Moreover, forced expression of lncRNA *HAR1A* attenuated the STAT3 pathway, whereas depletion of the same lncRNA upregulated the activity of this pathway. More importantly, rescue experiments confirmed that the knockdown of lncRNA *HAR1A* enhanced NSCLC cell proliferation by activating the STAT3 pathway. JAK–STAT3 signaling pathway has been well documented for its functions in upregulating tumor cell proliferation, survival, tumor invasion, angiogenesis, and immunosuppression [35]. STAT3 mainly acts as a direct transcription factor. Upstream activation signals lead to the phosphorylation of STAT3 at Tyr705 and the formation of STAT3 dimers. The activated dimers transfer to the nucleus and are recruited to the promoters of targeted genes to initiate transcriptional processes, consequently triggering diverse cellular events related to cancer progression [35,36]. Many lncRNAs have been reported to promote tumor growth through STAT3 in NSCLC [37,38,39]. LINC81507 was found to enhance NSCLC progression and metastasis by competitively sponging miR-199b-5p to provoke the CAV1/STAT3 pathway [38]. Similarly, lncRNA H19 promotes NSCLC development by regulating the miR-17/STAT3 axis [37]. Recently, Wu and colleagues demonstrated that lncRNAs LEISA accelerated the proliferation and inhibited apoptosis of lung adenocarcinoma cells in vitro and in vivo by facilitating STAT3 to transcriptionally activate IL-6 [39]. These data suggest that the STAT3 pathway is implicated in the lncRNA *HAR1A*-mediated effects on proliferation and apoptosis of lung cancer cells. 

Moreover, our results showed that *HAR1A* was downregulated in NSCLC. Interestingly, *HAR1A* was differentially expressed in the tested NSCLC cell lines. The upstream mechanisms regulating the expression of *HAR1A* deserve investigation in the future, including, but not limited to, encoding gene mutation, promoter methylation, transcriptional regulation, RNA m^6^A modification, RNA splicing, and RNA stability. Moreover, it is interesting to compare malignant potential between NCI-H1975 with high *HAR1A* expression and cell lines (A549, PLA-801D, and NCI-H292) with low HAR1A expression. 

## 5. Conclusions

Our findings indicated that lncRNA *HAR1A* was decreased in tumors and negatively associated with clinical outcomes in NSCLC. The gene signature of lncRNA *HAR1A*-related cancer cell survival genes was predictive of prognosis and sensitivity to anti-tumor drugs. Moreover, overexpression of lncRNA *HAR1A* inhibited NSCLC cell proliferation in vivo and in vivo by regulating the phosphorylation of STAT3. lncRNA *HAR1A* may be a novel therapeutic target for NSCLC. Further investigations are warranted to validate the prognostic value of this gene signature as biomarkers of OS in different patient cohorts using freshly dissected tumor tissues.

## Figures and Tables

**Table 1:** Coefficients of genes in the risk signature.

Gene	Coefficients
CASP9	0.022260317
BIRC3	0.170892327
IL1A	0.072875772
GPX3	−0.0695151
BTG2	−0.04849034
HGF	−0.115076855
SOD1	0.187145826
IER3	0.032801863
BCL2L10	0.076058979
CD2	−0.104266185
PSEN1	0.045078508
CD69	−0.017472337
BCL2L1	0.025612127
MGMT	−0.133525302
F2	0.031000311
NEDD9	−0.049292416
PDCD4	−0.077700047
ABL1	0.233491515
PTTG1	0.009826465
CDC25C	0.056608252
YWHAZ	0.163054991
YWHAE	0.149670734
ANAPC4	−0.341219736

## Data Availability

The datasets used and analyzed during the current study are available from the corresponding author on reasonable request. Published data analyzed in this study can be found in the following: the TCGA database (https://www.cancer.gov/about-nci/organization/ccg/research/structural-genomics/tcga. accessed on 28 October 2020), the CCLE database (https://sites.broadinstitute.org/ccle. accessed on 14 November 2020), and Molecular Signatures Database v7.1 (MSigDB, https://www.gsea-msigdb.org/gsea/index.jsp. accessed on 18 November 2020).

## Supplementary Materials

The following supporting information can be downloaded at: https://www.mdpi.com/article/10.3390/cancers14122845/s1, Figure S1: Decreased expression of lncRNA *HAR1A* in NSCLC; Figure S2: Western blot analysis was used to examine the expression of apoptotic (BAX and BCL-2) and proliferative (MCM2 and PCNA) biomarkers in NSCLC cells.; Figure S3: The effects of HAR1A on the STAT3 signaling pathway in NSCLC cells.; Figure S4: STAT3 inhibitor abolished the activation of the STAT3 signaling pathway caused by silencing HAR1A in NSCLC cells.
