# Supplementary material for: LncRNA HAR1A Suppresses the Development of Non-Small Cell Lung Cancer by Inactivating the STAT3 Pathway
Source: Cancers (Basel). 2022 Jun 8;14(12):2845. doi: 10.3390/cancers14122845 (PMC9221461; doi:10.3390/cancers14122845)
Supplement: Supplementary file 1 [file cancers-14-02845-s001.zip › cancers-1693837-supplementary.pdf]

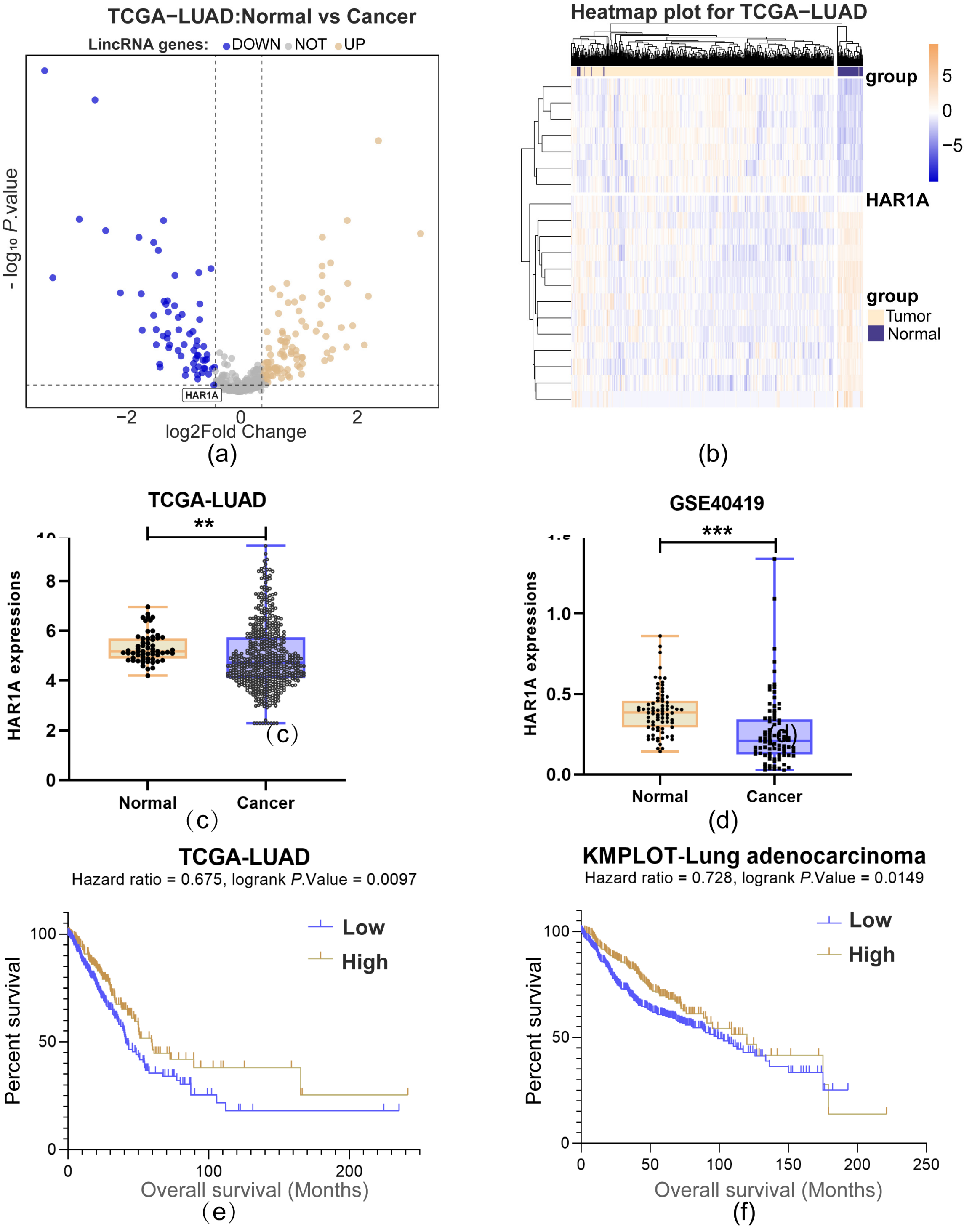

Figure S1: Decreased expression of lncRNA *HAR1A* in NSCLC. (a) Volcano plot of differently expressed lincRNAs in TCGA-LUAD. (b) Heatmap of up-and downregulated lincRNAs in TCGA-LUAD. LncRNA *HAR1A* was significantly decreased in tumors compared to normal tissues in TCGA-LUAD (c) and GSE40419 cohorts (c). The expression of HAR1A was negatively associated with the overall survival in the TCGA-LUAD cohort (e) and LUAD patients from KM Plotter (f). **, $P<0.01$, ***, $P<0.001$.

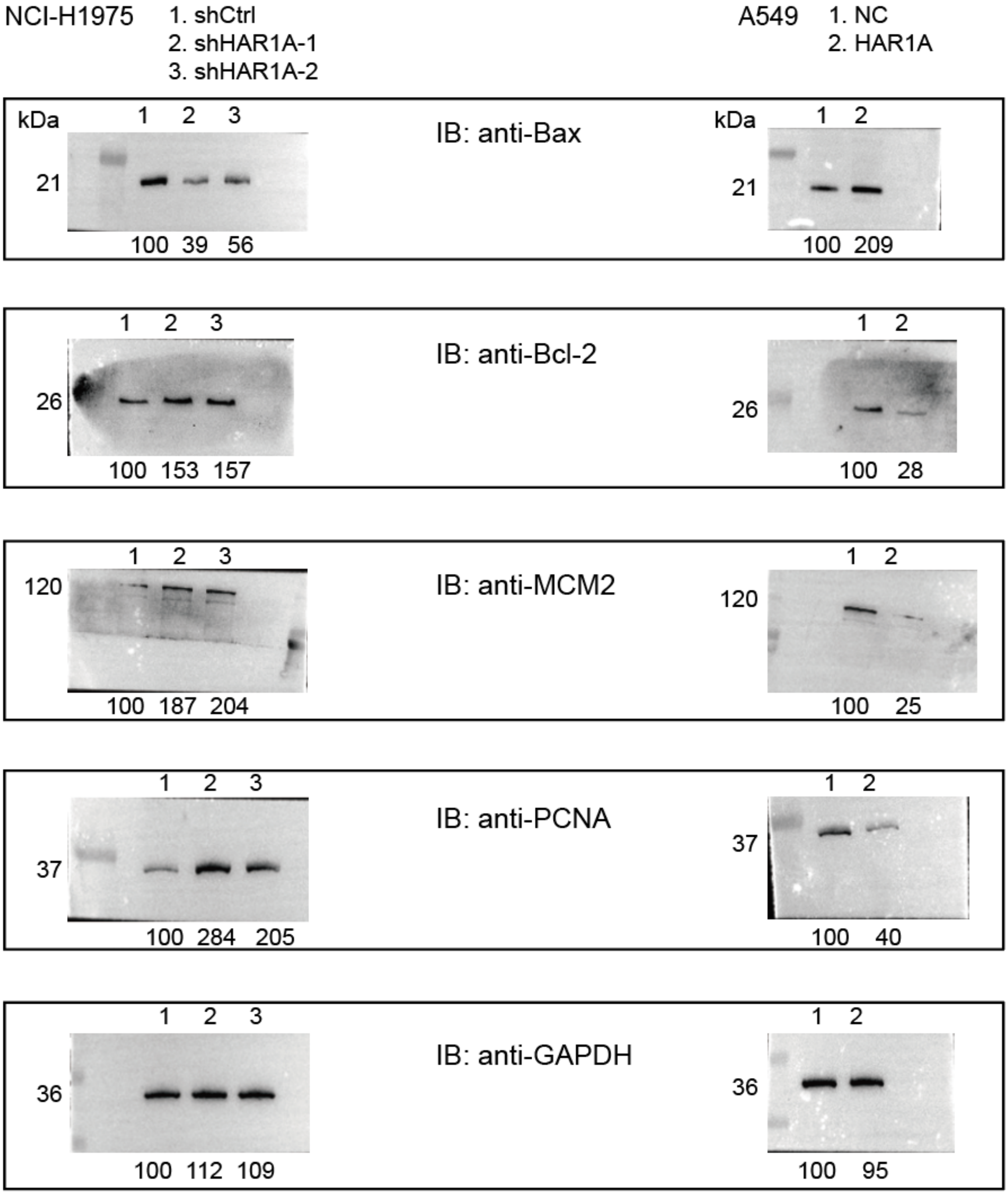

Figure S2: Western blot analysis was used to examine the expression of apoptotic (BAX and BCL-2) and proliferative (MCM2 and PCNA) biomarkers in NSCLC cells.

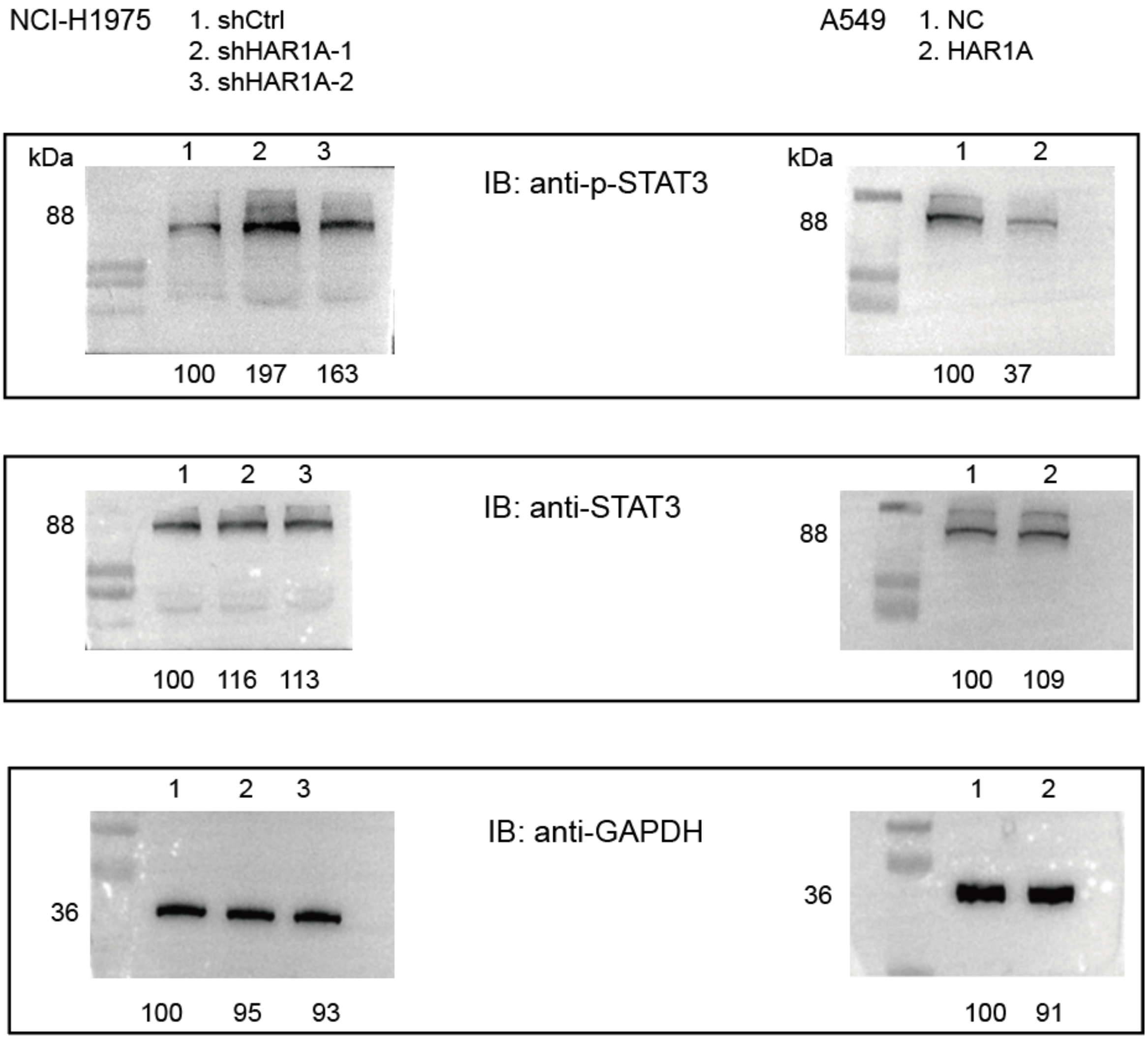

Figure S3: The effects of HAR1A on the STAT3 signaling pathway in NSCLC cells.

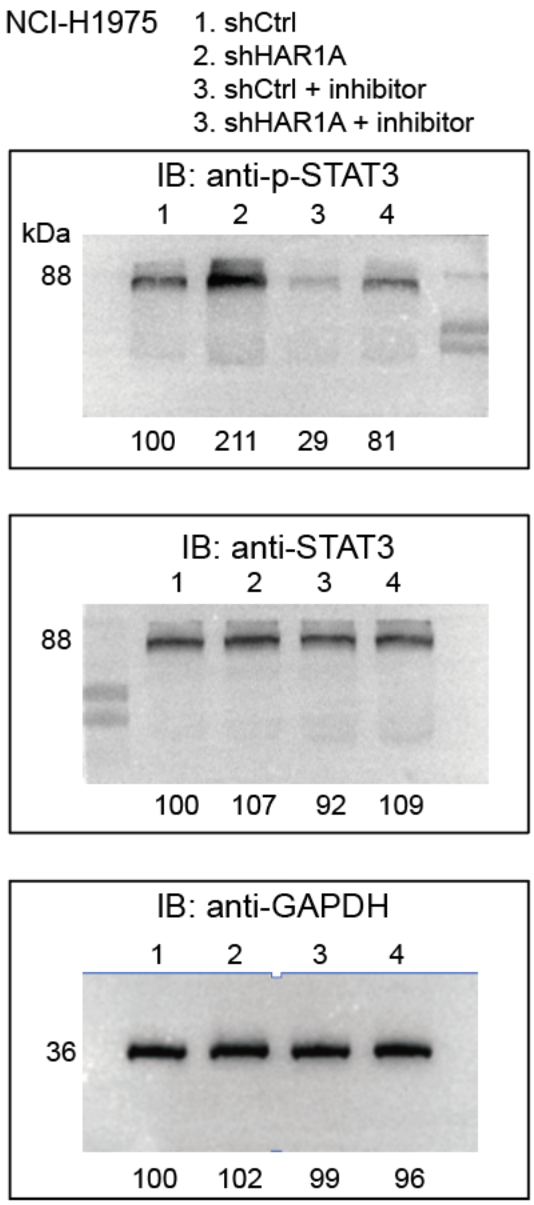

Figure S4: STAT3 inhibitor abolished the activation of the STAT3 signaling pathway caused by silencing HAR1A in NSCLC cells.
